# Residual-Shuffle Network with Spatial Pyramid Pooling Module for COVID-19 Screening

**DOI:** 10.3390/diagnostics11081497

**Published:** 2021-08-19

**Authors:** Mohd Asyraf Zulkifley, Siti Raihanah Abdani, Nuraisyah Hani Zulkifley, Mohamad Ibrani Shahrimin

**Affiliations:** 1 Department of Electrical, Electronic and Systems Engineering, Faculty of Engineering and Built Environment, Universiti Kebangsaan Malaysia, Bangi 43600, Selangor, Malaysia; 2 Faculty of Humanities, Management and Science, Universiti Putra Malaysia Bintulu Campus, Bintulu 97008, Sarawak, Malaysia; sitiraihanah.abdani@gmail.com (S.R.A.); ibrani@upm.edu.my (M.I.S.); 3 Community Health Department, Faculty of Medicine and Health Sciences, Universiti Putra Malaysia, Serdang 43400, Selangor, Malaysia; GS52834@student.upm.edu.my

**Keywords:** COVID-19 screening, X-ray imaging, convolutional neural networks, lightweight model

## Abstract

Since the start of the COVID-19 pandemic at the end of 2019, more than 170 million patients have been infected with the virus that has resulted in more than 3.8 million deaths all over the world. This disease is easily spreadable from one person to another even with minimal contact, even more for the latest mutations that are more deadly than its predecessor. Hence, COVID-19 needs to be diagnosed as early as possible to minimize the risk of spreading among the community. However, the laboratory results on the approved diagnosis method by the World Health Organization, the reverse transcription-polymerase chain reaction test, takes around a day to be processed, where a longer period is observed in the developing countries. Therefore, a fast screening method that is based on existing facilities should be developed to complement this diagnosis test, so that a suspected patient can be isolated in a quarantine center. In line with this motivation, deep learning techniques were explored to provide an automated COVID-19 screening system based on X-ray imaging. This imaging modality is chosen because of its low-cost procedures that are widely available even in many small clinics. A new convolutional neural network (CNN) model is proposed instead of utilizing pre-trained networks of the existing models. The proposed network, Residual-Shuffle-Net, comprises four stacks of the residual-shuffle unit followed by a spatial pyramid pooling (SPP) unit. The architecture of the residual-shuffle unit follows an hourglass design with reduced convolution filter size in the middle layer, where a shuffle operation is performed right after the split branches have been concatenated back. Shuffle operation forces the network to learn multiple sets of features relationship across various channels instead of a set of global features. The SPP unit, which is placed at the end of the network, allows the model to learn multi-scale features that are crucial to distinguish between the COVID-19 and other types of pneumonia cases. The proposed network is benchmarked with 12 other state-of-the-art CNN models that have been designed and tuned specially for COVID-19 detection. The experimental results show that the Residual-Shuffle-Net produced the best performance in terms of accuracy and specificity metrics with 0.97390 and 0.98695, respectively. The model is also considered as a lightweight model with slightly more than 2 million parameters, which makes it suitable for mobile-based applications. For future work, an attention mechanism can be integrated to target certain regions of interest in the X-ray images that are deemed to be more informative for COVID-19 diagnosis.

## 1. Introduction

Even after a year since the start of the Coronavirus disease 2019 (COVID-19) pandemic, there are still many countries that struggle with the increased number of COVID-19 cases everyday [1]. Moreover, there are several new COVID-19 mutations that are more prevalent among the younger peoples that have a higher infection rate [2]. Luckily, several of the developed vaccines like AstraZeneca, Pfizer, and Moderna have been proven to work well in reducing the number of infected cases even for these new COVID-19 variants [3]. Thus, the screening task of the COVID-19 cases is becoming more important so that high-risk patients can be identified immediately, which is a crucial step in breaking the infection chain of the virus. These patients were then to be quarantined in dedicated centers, whereby patients with severe symptoms need to be admitted to the hospitals for further intensive treatment. According to the World Health Organization, the suggested diagnosis method to detect this severe acute respiratory syndrome coronavirus 2, also known as SARS-CoV-2, which is the cause for the COVID-19 disease is through a reverse transcription-polymerase chain reaction (RT-PCR) test [4]. However, the cost of the RT-PCR test can be considered as expensive for most of the developing countries, whereby their economies have also been affected heavily by this pandemic. Hence, an effective screening method that can detect the disease immediately such as a rapid antigen test has been proposed to screen the patient with a high likelihood to be positive of COVID-19 [5]. In general, the rapid antigen test is slightly less accurate with 90% sensitivity and hence the results still need to be confirmed with the RT-PCR test [6]. Nevertheless, the advantage of fast screening outweighs the danger of spreading the viruses caused by the late notification of SARS-CoV-2 infection.

**Figure 1:**
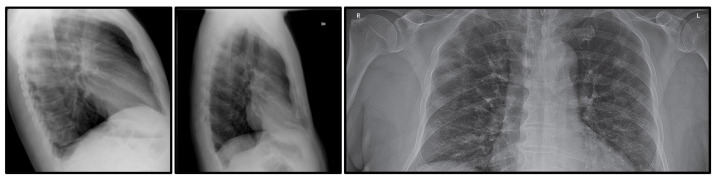
Samples of removed X-ray images. The first two samples are removed because they were captured from the side view, while the third sample is removed because of the incomplete information on the frontal chest X-ray image.

According to the current market price, a rapid antigen test costs around 30 USD, and it is still a pricey cost in the case of mass testing, especially in the industrial settings [7]. Therefore, an automated X-ray based screening method has been proposed to offer a low-cost screening alternative, while still producing fast screening results. The cost of an X-ray procedure is around one-fourth of the rapid antigen test and the machine is widely available all over the world [8]. Therefore, no additional specific equipment is needed, whereby small clinics are also known to have an X-ray machine. However, this imaging procedure needs to be complement with an automated screening method since not all health practitioners are well versed in the COVID-19 detection [9]. An X-ray-based screening requires a lot of prior experience, where the general health practitioners need to familiarize themselves with the COVID-19 prognosis as seen from the chest X-ray image. In line with this argument, this paper proposes an automated screening algorithm to detect the likelihood of COVID-19 cases based on X-ray images using an advanced machine learning technique. The proposed system will only focus on full-frontal chest X-ray images; as such, any side or sliced frontal X-ray images as shown in Figure 1 will be removed from the dataset. This step is taken because of two-fold reasons, which are to limit the variation of the input images and to reduce inaccurate learning of the model due to noisy data in the COVID-19 class.

In this work, a lightweight convolutional neural network (CNN) is proposed using the Residual-Shuffle network concept, in which the main branch will be split into two sub-networks that will later be concatenated and shuffled together to better learn the features from various prior layers. A residual skip connection [10] is also added to reduce the possibility of a zero-gradient diminishing issue, where the full architecture of the proposed network consists of four stacks of the Residual-Shuffle unit. At the bottom layer, three parallel branches of a spatial pyramid pooling (SPP) unit [11] are also added to improve the network capability in handling multi-scale detection. The added unit will be useful for the detection of COVID-19 cases with various severity levels, whereby the size of the air pocket in the X-ray images will be different as the disease becomes more severe. The design of this SPP unit is in contrast to the simplified SPP network as used in [12], in which their multi-scale feature maps are directly obtained through repeated down-pooling without performing any convolution operation. Overall, the proposed architecture utilizes slightly more than 2 million parameters, which is a lot smaller than the upper threshold of a lightweight network as defined in [8]. Due to its lightweight nature, it can be implemented on various types of mobile platforms and still achieve acceptable processing speed. Therefore, the main novelties of the proposed Residual-Shuffle-Net are its lightweight and accurate CNN model with just ∼2 million parameters, and the residual-shuffle unit that allows better feature learning across several groups of channels. Moreover, the network has also been integrated with three parallel branches of an SPP unit to extract multi-scale features, which are crucial in distinguishing COVID-19 cases and other types of pneumonia cases. It is worth noting that the proposed method aims to be an early screening method, after which the patients still need to be diagnosed by qualified medical practitioners. Therefore, this method is more applicable to developing countries, whereby the cost of mass diagnosis is considerably high compared to the average wage rate. Besides that, due to the lack of good laboratory facilities in developing countries, the results of the RT-PCR test will usually come out a few days after the samples are taken, which makes the proposed fast screening method a good complimentary test.

This paper is organized into five sections, where a review of the CNN-based system in screening COVID-19 disease is summarized in Section 2. Section 3 discusses in detail the full architecture of the proposed Residual-Shuffle Network (Residual-Shuffle-Net). The source code for the Residual-Shuffle-Net can be found at https://github.com/asyrafzulkifley/Residual-Shuffle-Net/blob/main/Residual_Shuffle_Net%20Model (accessed on 1 August 2021). Section 4 provides details of the database used for the validation tests and the outputs of the experiments, whereby comprehensive discussions on their performance are elaborated with respect to the state-of-the-art benchmarked methods. Section 5 summarizes the limitations of the proposed work, while a concise section on conclusions and future works is proposed at the end of this paper.

## 2. Convolutional Neural Networks for COVID-19 Detection

Convolutional neural networks have been successfully applied in many applications such as video analytic [13], intelligent remote sensing [14], two-dimensional signal processing [15], biomedical diagnosis [16], and many more. The technology relies on a set of optimal features to represent a dedicated application, whereby the features are trained using large numbers of data, contrary to the handcrafted features in the standard machine learning approach. As an example of the standard machine learning approach, the work in [17] has extracted a set of handcrafted COVID-19 features as the input to a naive Bayes classifier. They have utilized textures and several morphological features to represent the possibility of COVID-19 cases. Moreover, there are two popular imaging modalities have been explored in COVID-19 detection, which are X-ray images and computed tomography (CT) scans. According to Sverzellati et al. [18], X-ray is the better imaging modality compared to the CT scan in screening the possibility of COVID-19 cases, coupled with its low-cost procedures and wide availability of the X-ray machine. However, researchers in [19] found out that the segmentation of lung condition using CT scans produced a clearer mask for identifying the COVID-19 symptoms. In general, researchers have focused on either using a pre-trained model or developing a new dedicated model for the task of COVID-19 detection. This issue has been explored initially by Pham et al. [20], in which they have run several tests on the existing CNN models to verify the network’s effectiveness for the COVID-19 detection. They concluded that using pre-trained models of AlexNet, GoogleNet, and SqueezeNet were enough to achieve more than 98% accuracies on a three-class classification problem. However, there is an issue of class imbalance where they have used only 55 images of COVID-19 X-ray images compared to more than 1000 images for the other two classes.

Hence, several researchers have also explored basic pre-trained CNN such as the works by Pandit et al. [21] and Panwar et al. [22], who have retrained the VGG-16 architecture for a two-class problem to identify either COVID-19 or normal cases. Kikkisetti et al. [23] have also used the same VGG-16 architecture with minimal model modification, just by changing the last layer connection to a set of four nodes. Again, the transfer learning approach is used due to a limited number of datasets, whereby only 108 COVID-19 samples were used during training and testing phases. Apostolopoulos and Mpesiana [24] have experimented on five existing models for two and three-class classification problems, which are VGG-19 [25], MobileNet V2 [26], Inception [27], Xception [28], and Inception ResNet V2 [29]. They found out that the simplest VGG-19 model produced the highest accuracy. These results were obtained from limited numbers of training data with just 224 images of COVID-19 X-ray, whereby the other deeper models might experience under-fitting issues. Similarly, Narin et al. [9] have tested different sets of five CNN models, which are ResNet-50, ResNet-101, ResNet-152 [10], Inception V3 [30], and Inception ResNet V2 [29], and concluded that the simplest model ResNet-50 produces the highest accuracy. Again, only 341 X-ray images of COVID-19 patients were used during their experiments, which might point to the same underlying issue. Therefore, Loey et al. [31] have suggested the usage of a generative adversarial network (GAN) to create a set of synthetic images so that the training data for the COVID-19 class can be increased. A variety of GAN models have also been successfully applied to other applications such as traffic sign recognition [32], in which the synthetic training dataset has managed to improve their object detection accuracy. The work in [33] improvised on the GAN design, in which a conditional deep convolutional GAN is used to create the synthetic image separately for each class. Through this approach, more accurate and dedicated samples for the COVID-19 class were generated, since the other class data are more than enough for CNN training. In contrast, Ucar and Korkmaz [34] utilized a lightweight CNN model, SqueezeNet [35], that uses 2 million parameters to screen the COVID-19 cases. They have also optimized the hyper-parameters setup using Bayesian optimization. This optimization approach was also applied in [36], where an optimal set of hyper-parameters can boost the performance of the machine learning network.

On the other hand, Khan et al. [37] have modified the Xception network by changing the bottom layer with a flatten operator instead of a regular global average pooling operator. Even though Xception uses a separable convolution as its building block, the network runs comparatively slow due to its large number of parameters. Hence, Panahi et al. [38] have devised a fast network, FCOD, which requires just a total of 85,321 parameters. Their lightweight model has utilized a separable convolution format with a low number of convolution filter sets. Like the network by Khan et al., the work by Abdani et al. [8] has modified the bottom layer of SqueezeNet to integrate a spatial pyramid pooling unit for a three-class classification problem. They have argued that the air pockets in the X-ray images varied in size, especially between different severity levels of the COVID-19 cases. Hence, the CNN network must be able to capture these various scales of unique features, so that a robust COVID-19 identification system can be produced. Contrary to the parallel down-pooling unit in the SPP approach, Mahmud et al. [39] have introduced a network with several parallel atrous convolutions with different dilation rates. Thus, the captured receptive field size became bigger when the dilation rate was increased. They have also utilized a two-stage training, whereby the first stage focuses on the general classification of various pneumonia cases, and the second stage only focuses on differentiating between a COVID-19 case or not. The network introduced by Gilanie et al. [40] is unique in the sense that all convolution kernels are in the size of 5 × 5, which makes it less optimal for Tensorflow application. Their straightforward network consists of eight layers of convolution operator without applying any batch normalization technique. The CoroDet, which was introduced in [41], utilized a stack of seven down-sampling and up-sampling modules to produce a lightweight COVID-19 classifier, which has been extensively tested for two, three, and four-class classification problems. They have utilized a Leaky ReLU activation function, whereby the down-sampling operation is done through maximum down-pooling, while the up-sampling operation is done through feature map resize. These repeated down and up-sampling procedures were operated at small-size feature maps, which leads to a lot of information loss. Apart from COVID-19 detection, CNN has also been used to predict the severity level of the COVID-19 infection. In [42], a pre-trained VGG-16 architecture is used to predict the lung condition of the COVID-19 patients based on chest X-ray imaging. The transfer learning approach is used to initialize the network parameters, in which the last layer is modified to be a regressor node. Two different networks were trained for two different regression goals, which are to identify the geographic extent and degree of opacity of the lung. Similar to the previous work goals, Wong et al. [43] use a more complex network architecture of COVID-Net S to determine the severity level of the patient’s lung conditions. They have used the stratified Monte Carlo cross-validation method to further improve sampling strategy by grouping the chest X-ray images according to the age, sex, geographical location, imaging view, and imaging position attributes. They have also augmented their training dataset with image manipulation methods so that the dataset variability will be increased through translations, rotations, horizontal flips, zooms, intensity shifts, cutout, and Gaussian noise addition.

## 3. Residual-Shuffle Network with Spatial Pyramid Pooling Module

The Residual-Shuffle Network (Residual-Shuffle-Net) is a specialized lightweight CNN model built for COVID-19 detection for X-ray-based imaging systems. The network core component combines the residual skip connection with a compact shuffle unit, whereby a set of group convolutions is applied to let the network to learn multiple local features, instead of a set of global features. The design of this network is derived from the basic module of the ShuffleNet V2 [44], whereby the main branch is split into two branches at the beginning of the network and only one of the branches is treated with a convolution operator, while the other branch acts as a feed-forward unit. Generally, the Residual-Shuffle-Net consists of three modules of network flows, which are bottom, middle, and top modules. Let us define the Residual-Shuffle-Net, RS as the following with M representing the module and *I* representing the input image with the size of 256 × 256 pixels:

(1) $$\begin{array}{c} \mathrm{RS}=M_{1}.M_{2}.M_{3}.I \end{array}$$

The bottom module $M_{1}$ consists of an entry network that will extract rough features using two layers of convolution (C) and maximum down-pooling (P) operators. Each of the convolution operators applied in the Residual-Shuffle-Net will be followed by a batch normalization operator and a leaky rectified linear unit (Leaky ReLU) activation function. A small number of filter sizes is used for both convolution operations in the bottom network with just 8 and 16 channels to keep the number of parameters minimal during the early part of the network:

(2) $$\begin{array}{c} M_{1}=C_{1}.P_{1}.C_{2}.P_{2} \end{array}$$

The middle module $M_{2}$ comprises of the core Residual-Shuffle unit (RSU). There will be a stack of four sequential RSU, where P is added between them to down-scale the feature map size except for the fourth unit:

(3) $$\begin{array}{c} M_{2}=\left(\prod_{i=0}^{3}{\mathrm{RSU}}_{i}.P_{i}\right).{\mathrm{RSU}}_{4} \end{array}$$

**Figure 2:**
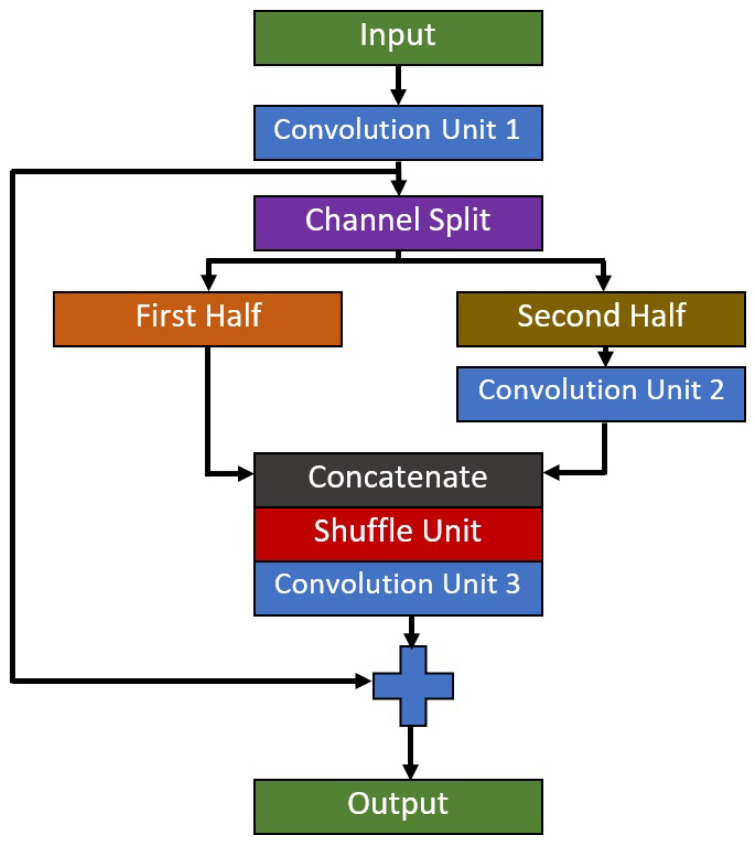
Architecture of the Residual-Shuffle unit.

The initial feature map size to this module is 64 × 64 pixels and the output feature map size at the end of this module is 8 × 8 pixels. Therefore, the range of the feature map size for this module is considerably small in order to maintain the lightweight nature of the proposed model. There will be three convolution operators in each RSU, where its full building block is shown in Figure 2. Residual-Shuffle-Net utilizes a bottleneck design for the convolution operations; as such, the middle convolution operator will have a smaller number of the filter set compared to the first and third convolution operators. The residual skip connection input layer comes from the output of the first convolution, which will be added to the output of the third convolution. Group and shuffle operations are performed during the second convolution operation, in which a simple channel split procedure is used to divide the feature maps into two equal groups, where it will be combined back using a concatenate operator. The addition of group and shuffle operations force the network to learn from various sets of feature branches, instead of one set of general features [45]. The design of sequential RSU allows the network to learn multiple local features instead of global features in each convolution layer.

**Figure 3:**
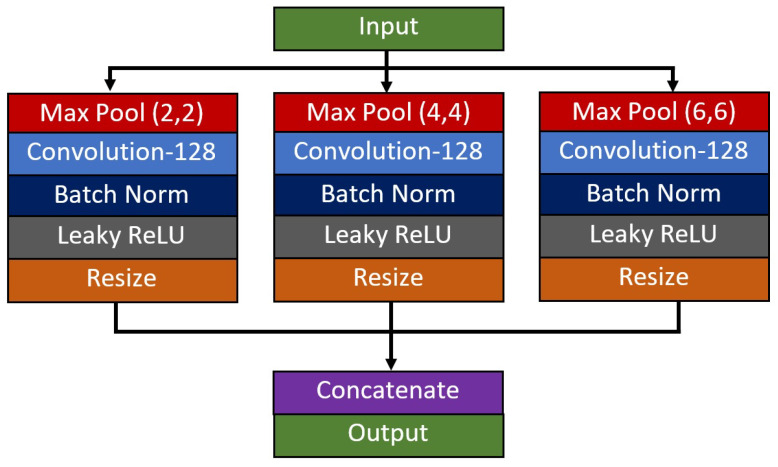
Architecture of the spatial pyramid pooling unit.

The final module $M_{3}$ utilizes a spatial pyramid pooling (SPP) unit, followed by a composite function of global average pooling and dense connection. Since this study focuses on a three-class problem, a SoftMax activation function with three output classes is used to come out with the likelihood of an image belong to each respective class. The SPP unit is added with the goal of improving the network’s capability in extracting multi-scale features of the diseases from X-ray images. In this paper, the dataset used to validate the network performance consists of diseases from various severity levels of COVID-19. Thus, the size of the air pocket seen in the X-ray images varies in size, and some of them are difficult to identify and differentiate using naked eyes, especially for the case between COVID-19 and other types of pneumonia cases. In line with the previous reasoning, three parallel branches of down-pooling operators are employed to extract the multi-scale features, which will be resized and concatenated back at the end of the unit. Since the input feature map size is 8 × 8 pixels, a set of 2 by 2, 4 by 4, and 6 by 6 down-pooling kernels has been utilized to produce equally spaced down-pooling scales as shown in Figure 3.

Then, a standard global average pooling G operator is used to sample the best multi-scale features before the classification process is done using a dense feedforward layer. The full design of the Residual-Shuffle-Net architecture is given in Table 1, where each layer information is detailed out in terms of the filter size, kernel size, and stride step for all bottom, middle, and top modules. Let D represent the dense connection layer with three output classes, where a SoftMax activation function is applied to complete the Residual-Shuffle-Net classifier with a total of 2,090,491 parameters.

(4) $$\begin{array}{c} M_{3}=\mathrm{SPP}.G.D \end{array}$$

## 4. Experiments and Discussion

### 4.1. Dataset

**Figure 4:**
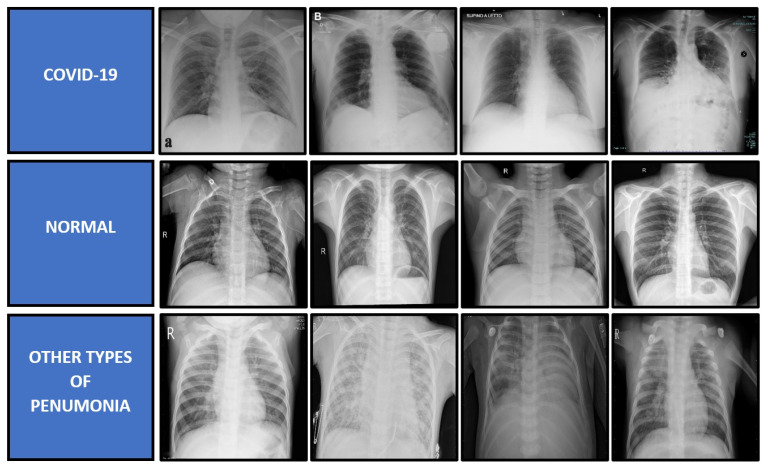
Samples of X-ray images for each category of COVID-19, normal and other types of pneumonia cases.

The proposed model is validated by using a three-class problem of X-ray image classification, which consists of COVID-19, other types of pneumonia, and normal cases. Due to the limited number of X-ray images for each category of bacterial and viral pneumonia, this study has chosen a three-class classification problem. Therefore, the class of other types of pneumonia contains both the virus and bacteria-caused pneumonia, except for COVID-19 cases for a fair deep learning comparison. The images for all three classes of X-ray radiography dataset were downloaded from two publicly available databases, which are Medical Imaging Databank of the Valencia Region (BIMCV) [46] and Radiological Society of North America (RSNA) [47]. The BIMCV dataset is the sole provider for the COVID-19 cases, while the RSNA dataset is the provider for the other types of pneumonia and normal cases. The dataset was skimmed through to select a set of quality images such that X-ray images with unrelated patterns or conditions will be removed. The main reason for this removal is to limit the possibility of the deep learning model in learning unrelated features to the disease such as color variation, background objects, side view images, and many more. If these images were not removed, these noisy patterns will be captured by the CNN model as parts of the disease patterns, which will produce an unfair comparison to the good X-ray imaging. In this case, the overall quality of the RSNA dataset is better than the BIMCV dataset. Besides that, even if the BIMCV dataset provides the radiological findings, this paper has omitted them as the respective findings are not available for the RSNA dataset. For the COVID-19 cases, all positive cases have also been confirmed with the RT-PCR test. The original mean age of the COVID-19 patient is 63 years old with a relatively fair distribution between the gender, with 46% of them being male and 54% of them being female. The dataset was captured from various types of X-ray machines, which were initially saved in Digital Imaging and Communications in Medicine (DICOM) format. According to the report in Vaya et al. [46], the majority of X-ray images were captured using fixed X-ray machines that include Konica Minolta 0862 342, GMM Accord DR 255, and Siemens FD-X X-ray machines. In the end, there are 1341 X-ray images for COVID-19 cases, 1341 images for other types of pneumonia cases, and 1341 images for normal cases, which sums up to 4023 X-ray images in total. All images are then saved in Portable Network Graphics (PNG) format with a standard resolution of 1024 × 1024 pixels, which is bigger than the input requirements of all benchmarked CNN models. Some samples of the X-ray images used in this paper are shown in Figure 4.

### 4.2. Evaluation Metrics

There are six evaluation metrics used to evaluate the performance of the proposed Residual-Shuffle-Net and its benchmarked methods. The selected metrics are accuracy $\left(\bar{ACC}\right)$, sensitivity $\left(\bar{SEN}\right)$, specificity $\left(\bar{SPE}\right)$, precision $\left(\bar{PRE}\right)$, F1-Score, and number of parameters. $\left(\bar{ACC}\right)$ concerns more on the true detection rate either for the positive or negative cases, while $\left(\bar{SEN}\right)$ and $\left(\bar{SPE}\right)$ concern more true positive rates and true negative rates, respectively. On the other hand, $\left(\bar{PRE}\right)$ measures the ratio of the correctly detected case and the total samples that have been predicted as positive, while F1-Score measures the harmonic mean between $\left(\bar{SEN}\right)$ and $\left(\bar{PRE}\right)$. All five of these metrics rely on basic units of true positive $\left(T_{Pos}\right)$, true negative $\left(T_{Neg}\right)$, false positive $\left(F_{Pos}\right)$, and false negative $\left(F_{Neg}\right)$. The $\left(F_{Pos}\right)$ and the $\left(F_{Neg}\right)$ are the cases when the predicted class does not match the labeled ground truth, whereby the prediction should have been positive and negative detection, respectively. On contrary, the $\left(T_{Pos}\right)$ and the $\left(T_{Neg}\right)$ are the cases when the predicted class exactly matches the ground truth label for both of the cases. Finally, the number of parameters represents the total number of trainable and non-trainable parameters utilized by the respective CNN model. The evaluation metrics are calculated as follows:

(5) $$\begin{array}{cc} \bar{ACC} & =\frac{T_{Pos}+T_{Neg}}{T_{Pos}+T_{Neg}+F_{Pos}+F_{Neg}} \end{array}$$

(6) $$\begin{array}{cc} \bar{SEN} & =\frac{T_{Pos}}{T_{Pos}+F_{Neg}} \end{array}$$

(7) $$\begin{array}{cc} \bar{SPE} & =\frac{T_{Neg}}{T_{Neg}+F_{Pos}} \end{array}$$

(8) $$\begin{array}{cc} \bar{PRE} & =\frac{T_{Pos}}{T_{Pos}+F_{Pos}} \end{array}$$

(9) $$\begin{array}{cc} F1-Score & =\frac{2T_{Pos}}{2T_{Pos}+F_{Pos}+F_{Neg}} \end{array}$$

### 4.3. Experimental Setup

There are 12 state-of-the-art CNN models from recent COVID-19 works that have been selected to be the performance benchmark for the proposed Residual-Shuffle-Net. All 12 models have utilized CNN classifiers for their COVID-19 detection system based on input from X-ray images, which are Hussain et al. [41], Abdani et al. [12], Khan et al. [37], Panahi et al. [38], Pandit et al. [21], Ozturk et al. [48], Mahmud et al. [39], Loey et al. [31], Ucar et al. [34], Panwar et al. [22], Narin et al. [9], and Gilanie et al. [40]. Five of the methods have used existing popular models, in which the models have been properly defined by the original authors, while the other seven methods have been selected because of their networks’ details were fully explained in their paper. All benchmarked models and the proposed Residual-Shuffle-Net have been coded on the Python platform using the Keras-Tensorflow library for a fair comparison, whereby their hyper-parameter settings have been tuned for the maximum classification performance. This is because the majority of the benchmarked papers have tested less than 600 images of COVID-19 cases. The main criterion used to judge for optimized hyperparameter settings for all methods is error convergence for training and validation loss function. The cutoff threshold value for both of the errors is 0.1, as such all models have been trained to produce errors less than the pre-set threshold value. Hence, a set of optimized settings as shown in Table 2 has been found using grid search methodology, whereby all the models achieved error convergence in their training and validation datasets. The performance metrics were also coded and analyzed using the Numpy library from Python software. One hot encoded labeling with SoftMax activation function is standardized as the last dense layer for all models. The Adam optimizer with a fixed learning rate is used to update the parameter values during the training phase, which has been set up to optimize the cross-entropy loss function with an accuracy performance metric. No simple or complex data augmentation was utilized, except for the image resizing operation to fit the input requirement of each model. In the Residual-Shuffle-Net case, the input image is resized to the resolution of 256 × 256 pixels. Batch size selection will depend on the model size, whereby the maximum possible batch size is used to train each of the models using a single Nvidia RTX 2080 Ti graphics card. Our Intel i9-9900K machine with a 3.60 GHz clock rate can afford to process Residual-Shuffle-Net with a batch size of 64 images. The proposed Residual-Shuffle-Net uses a total of 2,090,491 parameters, whereby 2,087,275 of them are trainable parameters, while 3216 of them are non-trainable parameters.

A five-fold cross-validation scheme is used to divide the dataset into general training and testing pots, so that the sampling bias can be reduced, whereas the over-fitting issue on the selected samples can be minimized. Then, the testing pot is further divided into two equal classes between validation and test dataset, whereby the final dataset is divided according to the ratio of 8:1:1 between training, validation, and testing phases. Therefore, for one validation fold, the number of X-ray images used for the training, validation, and testing are 3217, 403, and 403 images, respectively.

### 4.4. Discussion on the Residual-Shuffle-Net and Its Benchmarked Models Performance

**Figure 5:**
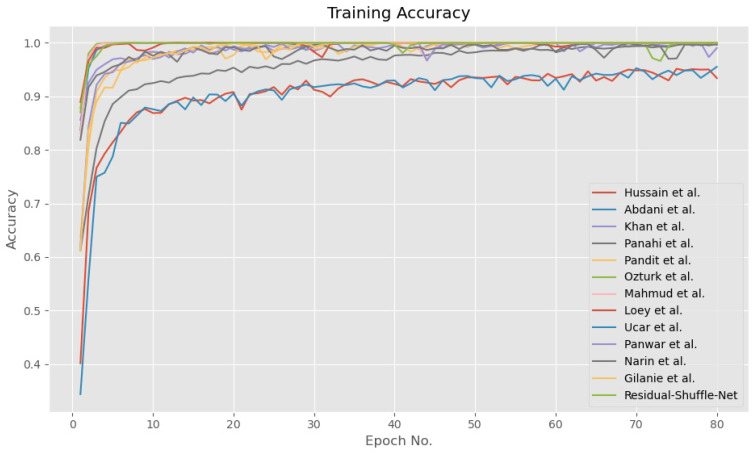
Training accuracy for Residual-Shuffle-Net and all of its benchmark methods.

**Figure 6:**
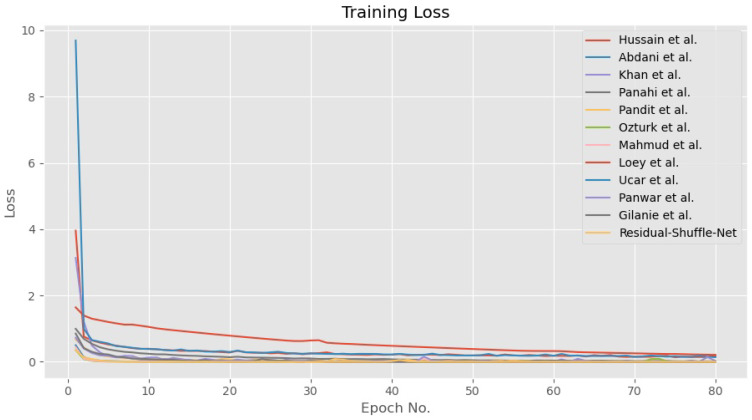
Training loss for Residual-Shuffle-Net and all of its benchmark methods.

**Figure 7:**
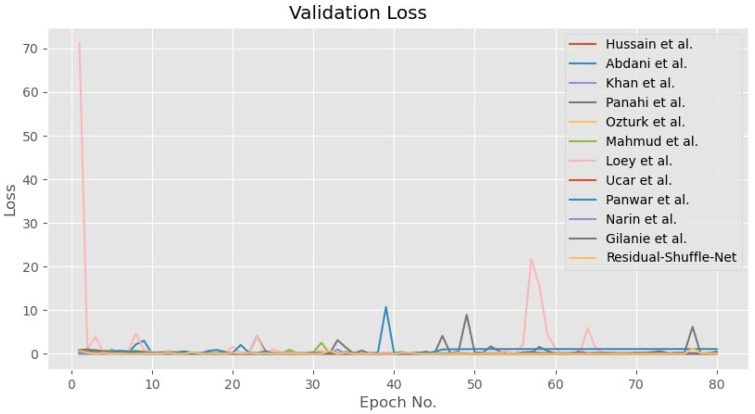
Validation loss for the Residual-Shuffle-Net and all of its benchmark methods.

In general, all the tested methods have been trained until convergence for both accuracy and loss functions as shown in Figure 5 and Figure 6, respectively. During the training phase, the error for all CNN models converged towards zero value, while the accuracy for 10 out of the 12 models converged towards maximum accuracy of 1.0. Besides that, the other two models by Panwar et al. [22] and Hussain et al. [41] have converged to 0.95 accuracies after 80 epochs of training update. The convergence pattern assumption is also supported by the validation loss curves as shown in Figure 7 that proved the Residual-Shuffle-Net and all the benchmark methods have been trained until convergence. The trend for both training and validation losses for all methods has converged towards zero error. Although there are six performance metrics were calculated in this study, two of them carry more weightage in determining the best detection method for COVID-19 screening. The goal of the screening stage is to detect as many true positive cases as possible that will be confirmed later by the RT-PCR test. Therefore, the $\bar{ACC}$ and $\bar{PRE}$ metrics were prioritized in ranking the benchmark methods, where both of them measure a certain ratio of positive cases over the total number of cases. However, it is worth noting that the false negative metric still plays an important role in COVID-19 screening. A screening method will be rendered useless if none of the cases were screened at the early stage, which will directly increase the cost of healthcare to the government. Table 3 shows the performance of the Residual-Shuffle-Net and its benchmark methods using all six performance metrics, which were ranked using $\bar{ACC}$ and $\bar{PRE}$. As a whole, Residual-Shuffle-Net performed the best in five out of the six evaluation metrics, except for the total number of parameters. It achieved the highest $\bar{ACC}$ and $\bar{PRE}$ with 0.97390 and 0.97403, respectively, while maintaining a relatively lightweight model with just 2 million parameters.

It is interesting to note that the second and the third-best CNN models, which are the methods by Gilanie et al. [40] and Abdani et al. [12], respectively, are both a specialized model designed for COVID-19 detection. The method by Gilanie et al. achieved an $\bar{ACC}$ of 0.96868, while the method by Abdani et al. achieved an $\bar{ACC}$ of 0.96395. Both of them can also be regarded as lightweight CNN models with total usage of parameters of less than 10 million. The uniqueness of the method by Gilanie et al. is in the selection of convolution kernel size, whereby 5 × 5 kernels were used throughout their network. A bigger kernel size can better capture the unique features on the X-ray images but comes with the main weakness of slower processing speed. On the other hand, Abdani et al. achieved good detection performance by relying on the simplified spatial pyramid pooling module that was able to capture multi-scale features of the X-ray images, which was crucial in distinguishing the cases of COVID-19 and other types of pneumonia. Their three parallel down-pooling branches did not consist of convolution operation, where the features maps were directly flattened for dense connections, which allows their network to maintain a small size of the total number of parameters.

The best pre-trained model performance among the benchmark models is returned by Khan et al. [37] method through the usage of Xception-71 architecture. They have slightly modified the top layer of the network to include a global average pooling operator instead of the original flatten operation. Their method managed to record an $\bar{ACC}$ of 0.96247 and a $\bar{SPE}$ of 0.98123 but requires a large model size of 88 million parameters, which is more than 42 times total number of parameters compared to the proposed Residual-Shuffle-Net. Even with a big-sized model, their model has utilized separable convolution schemes to reduce the demand of memory usage but its three-layer convolution unit still uses a large filter size of 728 channels. Surprisingly, a simple VGG-16 model, which was employed by Pandit et al. [21] and Panwar et al. [22] delivered the next best evaluation performance among the pre-trained models. Their architecture used 13 layers of the convolutional operation without utilizing any residual or feedforward branches. However, Panwar et al. modified the top layer of the network, by using a global average pooling operator, which resulted in much smaller model size, from the original 33 million parameters down to 14 million parameters. The three-layer dense connections in the original network used by Pandit et al. require a large number of parameters because there is a connection on each of the 4096 nodes, which also produced worse $\bar{ACC}$ of 0.94858 than the work by Panwar et al.

The overall worst performing method is recorded by the model designed by Hussain et al. [41]. Their model is relatively lightweight, in which they utilized repeated down-sampling and up-sampling processes on a small feature map size. The up-sampling operations that were applied to the small feature map size did not increase their network capability in extracting meaningful features from the X-ray images, which resulted in low $\bar{ACC}$ and F1-Score values of 0.78685 and 0.77785, respectively. However, the lowest F1-Score of 0.72718 was returned by the method of Narin et al. [9] that uses a pre-trained model of ResNet-50 architecture. Their low F1-score indicates that ResNet-50 produced a low ratio of true positive detection compared to the number of false detection, which makes it not suitable for the screening task. However, the method by Narin et al. produced a much higher $\bar{ACC}$ and $\bar{SPE}$ with 0.83765 and 0.88078, respectively, when compared to the method by Hussain et al. Contrary to that, a smaller version of ResNet-18 used by Loey et al. [31] managed to produce a better $\bar{ACC}$ of 0.94058 compared to the larger ResNet versions. Similar findings were also concluded in the work of Apostolopoilos and Mpesiana [24], where their simplest model among VGG-19, MobileNet V2, Inception, Xception-41, and Inception ResNet V2 produced the best COVID-19 detection. The reasoning behind these findings can be pointed towards small differences in COVID-19 features and other types of pneumonia cases, where the addition of compact multi-scale approach used in Residual-Shuffle-Net and method by Abdani et al. is more crucial to the classification performance rather than a deep residual connection network.

**Figure 8:**
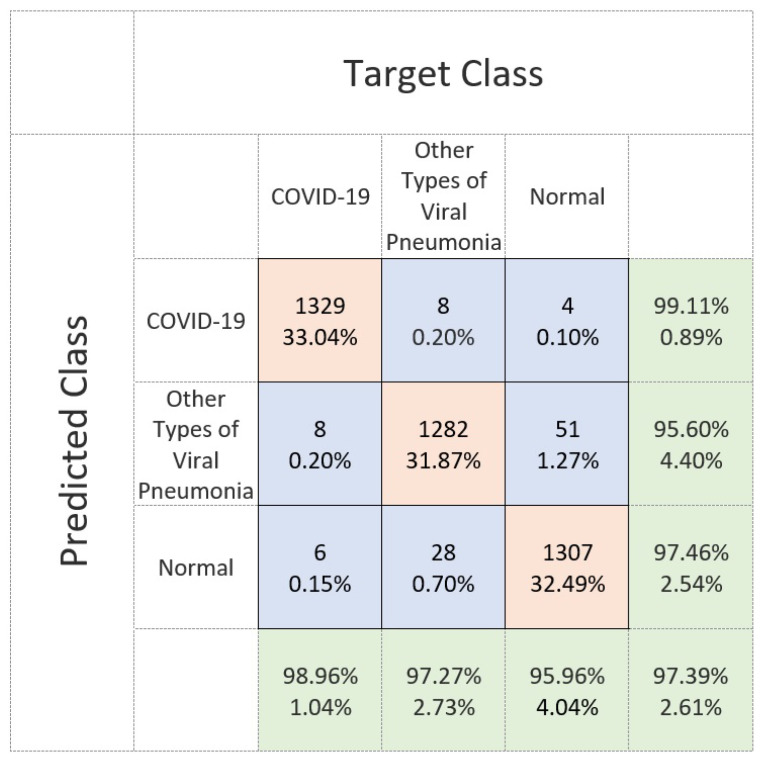
Confusion matrix performance of the Residual-Shuffle-Net in identifying the three classes of COVID-19, normal and other types of pneumonia cases using frontal chest X-ray images.

**Figure 9:**
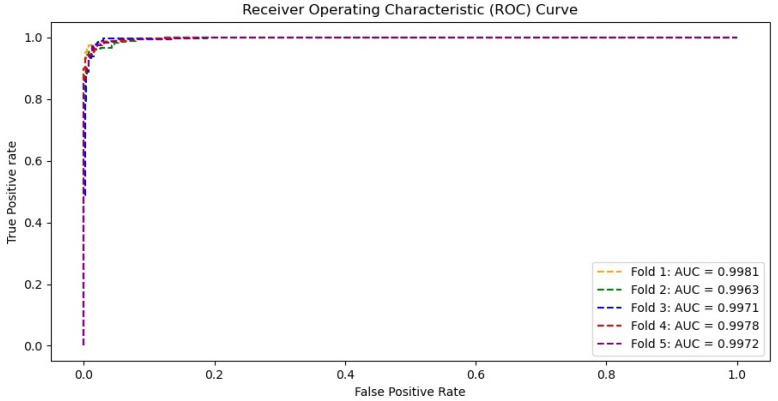
Receiver operating characteristic (ROC) curves for the Residual-Shuffle-Net with its respective area under the curve values.

Figure 8 shows the confusion matrix of the proposed Residual-Shuffle-Net in identifying the three classes of normal, COVID-19, and other types of pneumonia cases. This confusion matrix reports the performance of each class with respect to the other classes, instead of average performance information as shown in Table 3. The matrix provides the exact number of true positive, true negative, false positive, and false negative detections with regard to each of two other classes. The total number of samples on each class is uniform so that a fair comparison can be made to identify the weakness of the Residual-Shuffle-Net. The best true positive cases among the classes were recorded by the COVID-19 cases with 1329 out of 1341 X-ray images were correctly identified. Only eight cases of COVID-19 X-ray images were wrongly identified to be the other types of pneumonia cases. The main weakness of the proposed Residual-Shuffle-Net can be traced to false detection in the case of normal patients, where 51 normal cases were screened as other types of pneumonia cases. Similarly, 28 cases of other types of pneumonia were wrongly identified to be normal cases. One of the contributing factors behind this weakness is the quality of the X-ray images, whereby the datasets for normal and other types of pneumonia cases were captured in a more uniform setup. Contrary to that, X-ray images for COVID-19 cases were captured by using various machines with a different setup that leads to more variety in the imaging quality. Hence, it is easier to distinguish the COVID-19 cases compared to the other classes. However, the true positive detection for all classes remains high with the lowest case of 1282 true detections for the other types of pneumonia class, which is still a high accuracy with 95.6% true detection. For completion of the results, Figure 9 shows the receiver operating characteristic (ROC) curves for the proposed Residual-Shuffle-Net. The area under the curve (AUC) value for each validation fold is also provided, whereby the highest AUC of 0.9981 is achieved by the first fold, while the lowest AUC of 0.9963 is achieved by the second fold. Generally, the performance difference between the folds is very minimal with an AUC variance of 4.85 ×$10^{-7}$.

## 5. Limitations

There are few limitations of the proposed work, mainly due to the hardware limitation, emergence of new variants of concerns, and clinical test requirements. The core of the proposed method relies on the deep convolutional network that requires heavy computational power, especially during the training process. Therefore, an efficient computational platform is crucial during the training phase, while a lesser intensive computation is needed during the testing or screening phase. Moreover, the proposed deep network cannot distinguish the various types of the COVID-19 mutation, especially with regard to the recent variants of concern such as delta and lambda variants. This is because most of the existing X-ray images were taken from the early variants of COVID-19, whereby images of the newer mutations are still being added continuously to the dataset. Besides this, the proposed method still requires confirmation diagnosis from the medical practitioners. This approach is still in the early phase of development, whereby more clinical testings need to be performed before it is suitable for mass usage.

## 6. Conclusions

In conclusion, this study managed to prove the effectiveness of the Residual-Shuffle-Net in detecting COVID-19 cases based on the X-ray imaging input. The main novelty of the proposed network lies in its lightweight residual-shuffle unit that combines the split and shuffle unit with a residual skip connection. This architecture allows the network to better learn the distinguishing features between the COVID-19 cases and other class categories. In addition, the network is also embedded with a spatial pyramid pooling unit that enables it to extract multi-scale features, which is important for detecting COVID-19 cases of various severity levels. The Residual-Shuffle-Net returned the best performance for five performance metrics, which are $\bar{ACC}$, $\bar{SEN}$, $\bar{SPE}$, $\bar{PRE}$, and F1-Score with 0.97390, 0.97390, 0.98695, 0.97403, and 0.97387, respectively. Although the method by Panahi et al. uses the lowest total number of parameters, Residual-Shuffle-Net is still considered as a lightweight model with just 2,090,491 parameters. The classification performance can be further improved by considering an attention mechanism that allows the network to focus on selected regions of interest, rather than treating the whole image as equal. Besides this, a separable convolution approach can also be implemented to reduce memory usage.

## Figures and Tables

**Table 1:** Overall architecture of the Residual-Shuffle-Net.

No.	Layer	Output Size	Filter Size	Kernel Size	Stride
1	Convolution	256 × 256	8	3 × 3	1 × 1
2	Max Pooling	128 × 128	-	2 × 2	2 × 2
3	Convolution	128 × 128	16	3 × 3	1 × 1
4	Max Pooling	64 × 64	-	2 × 2	2 × 2
5	Residual-Shuffle (1)	64 × 64	32-16-32	3 × 3 − 1 × 1 − 3 × 3	1 × 1
6	Max Pooling	32 × 32	-	2 × 2	2 × 2
7	Residual-Shuffle (2)	32 × 32	64-32-64	3 × 3 − 1 × 1 − 3 × 3	1 × 1
8	Max Pooling	16 × 16	-	2 × 2	2 × 2
9	Residual-Shuffle (3)	16 × 16	128-64-128	3 × 3 − 1 × 1 − 3 × 3	1 × 1
10	Max Pooling	8 × 8	-	2 × 2	2 × 2
11	Residual-Shuffle (4)	8 × 8	256-128-256	3 × 3 − 1 × 1 − 3 × 3	1 × 1
12	Spatial Pyramid Pooling	8 × 8	128-128-128	2 × 2 − 4 × 4 − 6 × 6	1 × 1
13	Global Average Pooling	1 × 1	-	8 × 8	1 × 1
14	Dense + SoftMax	-	3	1 × 1	1 × 1

**Table 2:** Hyper-parameter settings for the Residual-Shuffle-Net.

Criteria	Hyper-Parameter Setting
Batch size	64
Training epoch	80
Backpropagation method	Adam optimizer
Input image size	256 × 256 pixels
Optimizer learning rate	0.0001
Optimizer momentums	$\beta_{1}$ = 0.9, $\beta_{2}$ = 0.999
Loss function	categorical cross-entropy
Labeling format	One-hot encoded

**Table 3:** Performance results of the Residual-Shuffle-Net and the benchmark methods.

Method	$\bar{\mathrm{ACC}}$	$\bar{\mathrm{SEN}}$	$\bar{\mathrm{SPE}}$	$\bar{\mathrm{PRE}}$	F1−Score	Total Parameters	Trainable Parameters
Hussain et al. [41]	0.78695	0.78695	0.89347	0.81998	0.77785	3,798,083	3,798,083
Narin et al. [9]	0.83765	0.76155	0.88078	0.8376	0.72718	23,567,299	23,514,179
Ozturk et al. [48]	0.84629	0.84629	0.92318	0.92572	0.81911	1,167,363	1,164,143
Ucar et al. [34]	0.92841	0.92841	0.96420	0.93130	0.92866	1,078,211	1,078,211
Mahmud et al. [39]	0.93686	0.93686	0.96843	0.94025	0.93609	1,338,291	1,164,143
Loey et al. [31]	0.94058	0.94058	0.97029	0.94387	0.94067	11,192,003	11,182,275
Panahi et al. [38]	0.94406	0.94406	0.97203	0.94674	0.94385	85,321	83,849
Pandit et al. [21]	0.94858	0.94858	0.97429	0.94859	0.94834	33,609,539	33,609,539
Panwar et al. [22].	0.95700	0.95700	0.978500	0.95746	0.95692	14,747,715	14,747,715
Khan et al. [37]	0.96247	0.96247	0.98123	0.96334	0.96247	88,054,139	87,958,763
Abdani et al. [8]	0.96395	0.96395	0.98197	0.96454	0.96388	1,862,331	859,883
Gilanie et al. [40]	0.96868	0.96868	0.98434	0.96884	0.96863	2,339,267	2,339,267
**Residual-Shuffle-Net**	**0.97390**	**0.97390**	**0.98695**	**0.97403**	**0.97387**	**2,090,491**	**2,087,275**

## Abbreviations

The following abbreviations are used in this manuscript:

SPP	Spatial Pyramid Pooling
CNN	Convolutional Neural Networks
COVID-19	Coronavirus Disease 2019
RT-PCR	Reverse Transcription Polymerase Chain Reaction
CT	Computed Tomography
GAN	Generative Adversarial Network
Residual-Shuffle-Net	Residual-Shuffle Network
Leaky ReLU	Leaky Rectified Linear Unit
BIMCV	Medical Imaging Databank of the Valencia Region
RSNA	Radiological Society of North America
DICOM	Digital Imaging and Communications in Medicine
PNG	Portable Network Graphics
ROC	Receiver Operating Characteristic
